# Characterization and technical evaluation of physicochemical properties of Tibetan medicine Zuotai via modern processing

**DOI:** 10.3389/fphar.2026.1900757

**Published:** 2026-10-14

**Authors:** Renzhen Wangjia, Gaden Nyima, Kelsang Gyab Ala, Zhaxi Cuo, Danba Gyatso

**Affiliations:** 1 School of Pharmacy, Qinghai Minzu University, Xining, Qinghai, China; 2 Men Tsee Khang, Tibetan Medical Hospital of Tibet Autonomous Region, Lhasa, China; 3 School of Chemistry and Material Science, Qinghai Minzu University, Xining, China; 4 Gannan Prefecture Hezuo City Kajiaman Tibetan Medicine Development Co., Ltd., Gannan, China

**Keywords:** Tibetan medicine, Zuotai, mineral medicine processing, modern processing, physicochemical analysis

## Abstract

Zuotai, known as the “king of medicines” in Tibetan pharmacology, is a crucial ingredient in precious formulations such as Seventy-flavor Pearl Pills. Traditional preparation of Zuotai is complex, time-consuming, labor-intensive, and poses significant toxicity risks. To address these challenges, our research team has modernized the process by replacing traditional tools with jacketed reactors and controlled heating, significantly reducing toxicity, shortening processing time from over 40 days to about 20 days, and enhancing reproducibility. In this study, five batches of Zuotai samples were characterized using X-ray fluorescence (XRF), scanning electron microscopy (SEM), X-ray diffraction (XRD), and Fourier-transform infrared spectroscopy (FT-IR). Results confirmed consistent physicochemical properties across all batches, dominated by HgS with excess sulfur. Safety evaluation via *in vitro* simulated gastric/intestinal fluid dissolution tests demonstrated that the soluble mercury release from modern-processed Zuotai was only 38.64 μg/g (0.0258% of total Hg) under the most stringent conditions (SGF with 10 mM L-cysteine, 37 °C, 24 h), corresponding to a daily intake of approximately 7.73 μg—only 18% of the FAO/WHO acceptable daily intake (43 μg/day), offering a standardized approach for Zuotai production.

## Graphical Abstract

**Figure:**
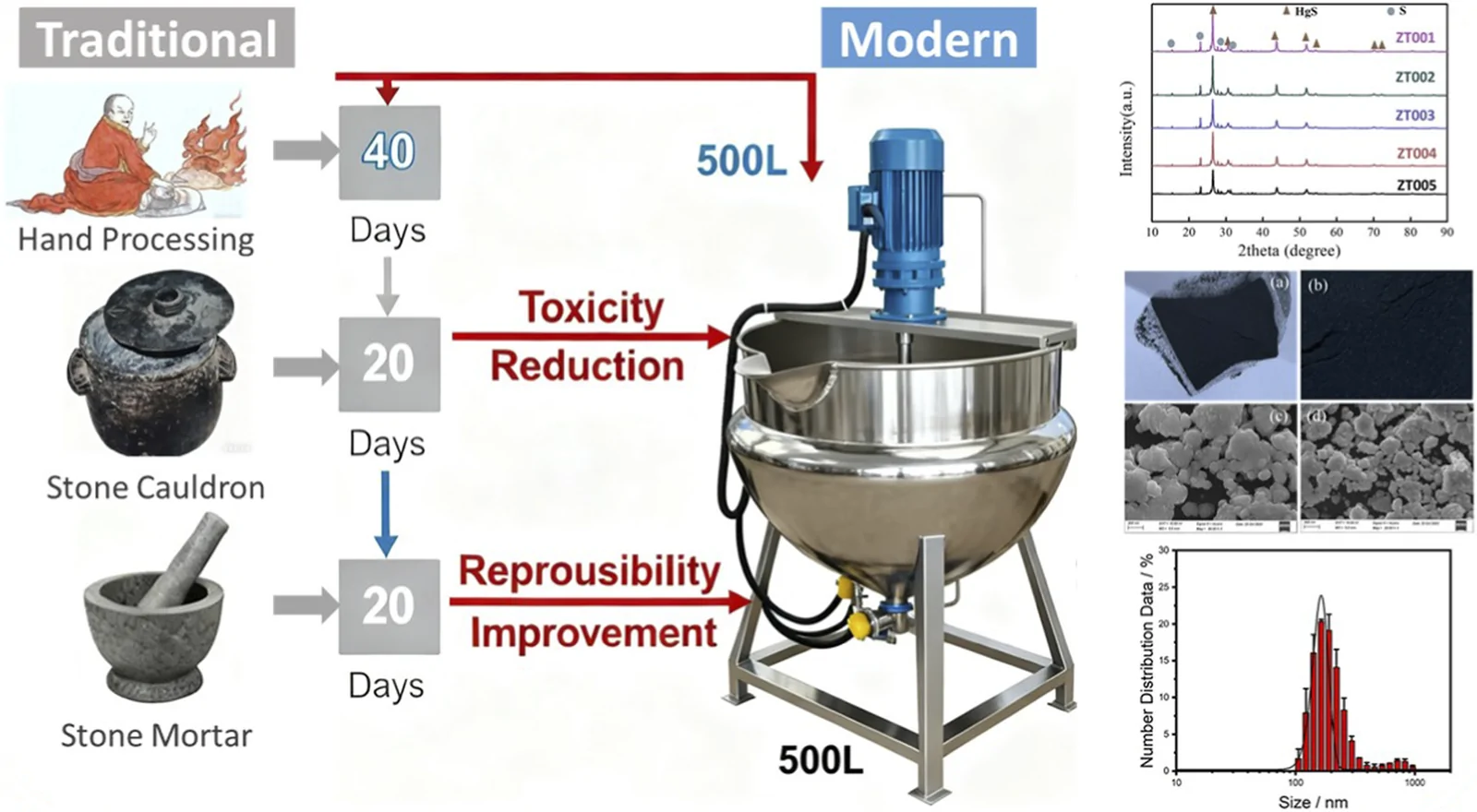

## Highlights

- Processing time was reduced by over 50% (from >40 days to ∼20 days) by modernizing the traditional method using a controlled jacketed reactor, significantly mitigating toxicity and health risks.
- Multi-technique characterization (XRD, SEM-EDS, XRF) of five batches confirmed highly consistent composition, dominated by well-crystallized HgS (≈15 wt% Hg) with excess sulfur (≈82 wt% S).
- The modern process demonstrates excellent reproducibility and scalability across different production sites, providing a standardized, safe, and efficient approach for industrial-scale Zuotai production.

## 1 Introduction

The processing technology of Tibetan medicine represents a historic achievement and essence of this medical system. It has formed a unique and independent system with distinctive characteristics, embodying profound cultural heritage and scientific significance, thus holding an important position in the field of world traditional medicine. Particularly, the processing of mineral medicines, represented by “Zuotai”, is the most remarkable and is considered the core technology of Tibetan medicine processing. With a history dating back over 2,600 years (Yutuo Yuandan, 1987; Luo, 2000; Luo, 1986), it has been included in the first batch of China’s National Intangible Cultural Heritage list (Chinese Intangible Cultural Heritage, 2008).

The term “Zuotai” is a transliteration from Tibetan: “Zuo” means decoction or boiling, and “Tai” means ash or charcoal, collectively referring to the ash obtained from processed mercury (He et al., 2015; Bowman and Russell, 2006). During its preparation, mercury is processed with more than 100 kinds of raw and auxiliary materials, including the “Eight Metals” and “Eight Minerals” (Cen et al., 2012; Yan et al., 2007). This involves over 300 complex procedures such as removal of impurities, detoxification, boiling, and calcination. The entire processing duration extends beyond 40 days, ultimately resulting in a black powdered form of Tibetan medicine known as “Zuotai” (Rang et al., 2025; Lang, 2007; Xiangyang et al., 2010). This process aims to eliminate the toxicity of mercury and alter its inherent properties. Zuotai is known for its detoxifying, tonic, and life-prolonging effects (Ga, 2005; JianCuo et al., 2025) and is commonly used in classic prescriptions for treating poisoning, gastrointestinal diseases, serious diseases (Tibetan: Huangshui disease), and complex diseases, and health maintenance (Cuo, 2001; Mingcheng et al., 2009; Zhang and Xiang, 2005). It is also an indispensable component in precious Tibetan medicinal formulations such as Seventy-flavor Pearl Pill, Renqing Changjue, Renqing Mangjue, and Zuozhu Daxi (Dun Man, 2011; Ni Ma, 2017). When combined with other formulations, it is believed to have a synergistic effect, significantly enhancing therapeutic efficacy.

Currently, the processing techniques for Tibetan medicine Zuotai vary across different regions and schools, leading to differences in the identification of raw/auxiliary materials and processing methods. As documented by Banma Cairen (Cairen et al., 2022), a survey of 21 Tibetan medical institutions across Qinghai, Gansu, Sichuan, and Tibet revealed significant variability in processing protocols, equipment, and quality control standards. This variation results in challenges in ensuring consistent quality, controllability, and safety, severely impacting the clinical application of Zuotai and the full realization of its therapeutic advantages. Furthermore, the tension between outdated industrial equipment, small production scale, and the increasing market demand coupled with high-quality requirements is becoming increasingly apparent. Consequently, innovation in the traditional manual processing techniques is urgently needed.

The presence of mercury in ethnic medicines has raised global public health concerns. The 2020 Chinese Pharmacopoeia (National Pharmacopoeia Commission, 2020) sets mercury limits for plant-based medicines at ≤0.2 mg/kg, with speciation analysis (HPLC-ICP-MS) required for mercury and arsenic. Internationally, the EMA (2022) (European Medicines Agency, 2022) and FDA (2016) (U.S. Food and Drug Administration, 2016) emphasize speciation-based risk assessment over total metal content. This approach is critical for Zuotai, whose safety depends on chemical form—with HgS being biologically inert. These requirements underscore the need for controllable detoxification technology that stabilizes mercury as HgS.

In recent years, several modernization attempts have been made to address the limitations of traditional Zuotai processing. According to the classification framework established by Qunpei (Qunpei et al., 2025), the current processing methods across Tibetan regions include four main approaches: (1) traditional manual processing (predominant in Sichuan Garzê, Tibet Qamdo, and Qinghai Yushu); (2) ball-mill mechanized processing (predominant in most areas of Qinghai); (3) traditional + ball-mill hybrid processing (used in Lhasa and Shannan of Tibet, Gannan of Gansu, and Aba of Sichuan); and (4) stirred jacketed reactor processing (used in Yunnan, Gansu, and Sichuan Garzê). While these modifications have reduced processing time to some extent, they still suffer from insufficient parameter quantification and lack of standardized quality control, resulting in inconsistent product quality across different production sites.

The modern Zuotai processing method presented in this study represents a novel approach that integrates specialized knowledge of Tibetan medicine with modern instrumentation. Professor Gawe’ (a representative inheritor of the first batch of National Intangible Cultural Heritage projects, specifically the compounding techniques of Renqing Changjue in Tibetan medicine) has effectively combined his extensive medical theory and years of practical experience with modern equipment (e.g., jacketed reactors). Through related chemical composition analyses and control of physical parameters lik e temperature and humidity, the previously difficult-to-master processing technique has been transformed into a controllable, reproducible, scalable, and more manageable conventional technology (Gyetsen Namdro et al., 2024). Compared to the traditional manual process, this modern approach significantly shortens the processing time, effectively reduces intoxication risks, and improves patient compliance (Nima, 2024; Wu et al., 2016). Table 1 compares the present jacketed reactor method with traditional manual processing and previously reported modification schemes. Unlike earlier attempts that focused on individual tool replacements, our approach achieves full-process parameter quantification—including temperature gradients, stirring speeds, and holding times—which is essential for industrial translation and regulatory compliance.

**TABLE 1:** Comparison of Zuotai processing methods: traditional, previously reported, and this work.

Parameter	Traditional manual processing	Previously reported modified methods	This work (jacketed reactor)
Core equipment	Leather bag, stone trough, stone mortar, iron pot, ceramic vessels	Ball mill, thermostatic stirring device	500 L electric heating jacketed reactor
Processing duration	Approximately 50–60 days (approx. 420 h) (Cairen et al., 2022; Qunpei et al., 2025)	Approximately 20–30 days (approx. 302 h) (Qunpei et al., 2025)	Approximately 20 days
Key process parameter control	Experience-dependent, qualitative control (“hand feeling, visual observation, taste testing, olfactory assessment”) (Cairen et al., 2022)	Partially quantified, still relies on operator experience	Fully quantified
Mercury vapor exposure risk	High (open system, direct operator contact with mercury) (Cairen et al., 2022)	Moderate (semi-enclosed, potential exposure remains)	Low (fully enclosed system with condenser and alkali scrubber)
Batch-to-batch consistency	Poor (operator-dependent, significant variation across different schools/traditions) (Cairen et al., 2022)	Moderate (insufficient equipment standardization)	High (CV < 12% across five batches; one-way ANOVA p > 0.05)
Single batch yield	Small (approx. 25 kg/batch)	Moderate	Large (>50 kg/batch)
Industrial scalability	Low	Moderate	High
Standardization level	Low (multiple schools, non-uniform methods) (Qunpei et al., 2025)	Moderate	High (controllable, reproducible, scalable)

This paper will briefly introduce the novel processing technology independently developed by Professor Gawe’s team. Furthermore, it will present physicochemical analyses conducted on five batches of Zuotai samples prepared by our team in relevant medical institutions within Tibetan-inhabited areas over the past 5 years. These analyses employed modern material characterization techniques including X-ray diffraction (XRD), scanning electron microscopy (SEM), fluorescence spectroscopy, and *in-situ* infrared spectroscopy. The study aims to investigate the safety, controllability, and innovativeness of the new process, thereby providing technical guidance for establishing standardization in Zuotai processing.

## 2 Experimental

### 2.1 Raw materials and equipment

Raw materials included “three acrid herbs”(dried ginger, pepper, piper longum), “salt-alkali” (blue salt, alkalinized substance), “eight Qiala” (borax water, ferrous sulfate water, salt water, loess water and four specific animal urines), “four additional auxiliaries” (garlic water, yak beef soup, butter, highland barley wine, etc.), “five fats” (animal fats from five sources), “three acids”(sea-buckthorn water, ferrous sulfate water, highland barley wine), “two oils”(rapeseed oil, castor oil), sulfur, “eight metals” (gold, silver, copper, iron and other four metals) as well as “eight minerals”(chalcopyrite, silver ore, pyrite, magnetite, sinter and so on). All raw and auxiliary materials were sourced from the pharmaceutical preparation departments of Tibetan medical institutions and authenticated by Professor Gawe’ (national-level representative inheritor). The “Eight Metals” and “Eight Minerals” were processed and calcined under his direct supervision to produce standardized Zuotai auxiliary materials, consistent with the Provincial Quality Standard of Qinghai and Sichuan, etc. The core processing equipment was a 500 L electric heating jacketed reactor.

### 2.2 Processing methodology

All processing parameters were quantitatively controlled in the jacketed reactor system. The detailed operational parameters for each stage are summarized in Table 2. As documented in the literature, traditional manual processing requires approximately 420 h (about 60 days) to complete (Qunpei et al., 2025). In comparison, our modern jacketed reactor method, with full parameter quantification, reduces the processing time to approximately 20 days, representing a significant improvement in efficiency while maintaining strict process control. The complete processing sequence, including feeding order, temperature profiles, and holding times, was designed to follow traditional protocols while enabling precise parameter control for reproducibility and scalability. The detailed processing methodology is summarized in the technical flowchart presented in Figure 1. The procedure is briefly described as follows: Mercury was initially subjected to a series of purification and detoxification steps. This involved rigorously rubbing the mercury with specific auxiliary ingredients to remove surface impurities, followed by sequential washing procedures with detoxifying agents to eliminate inherent toxicity. Subsequent to the primary detoxification, an additional supplementary detoxification step was carried out to ensure complete removal of toxic elements.

**TABLE 2:** Key process parameters of the jacketed reactor method for Zuotai processing.

Parameter category	Specific parameter	Value/Setting
Reactor settings	Stirring speed	80–120 rpm (throughout all operations)
​	Heating method	Electric heating jacketed reactor with programmable temperature control
Detoxification stage	Dry detoxification (three acrid powders)	Room temperature, 48 h with stirring/kneading
​	Wet detoxification (salt-alkali solutions)	Sequential addition of various steeping solutions, 96 h with stirring
Boiling/calcination stages	Major boiling	100 °C, 92 h
​	Medium boiling	100 °C, 48 h
​	Minor boiling	150 °C, 24 h
Mixing stage	Stirring speed/temperature	80 rpm, <100 °C, 12 h
Feeding	Sulfur:mercury ratio	1:1 (w/w)
​	Feeding sequence	Sequential addition following traditional protocol order
​	Eight metals/eight minerals ash addition	Added after small boiling, during mixing stage
Total processing time	Complete process	Approximately 20 days

**FIGURE 1:**
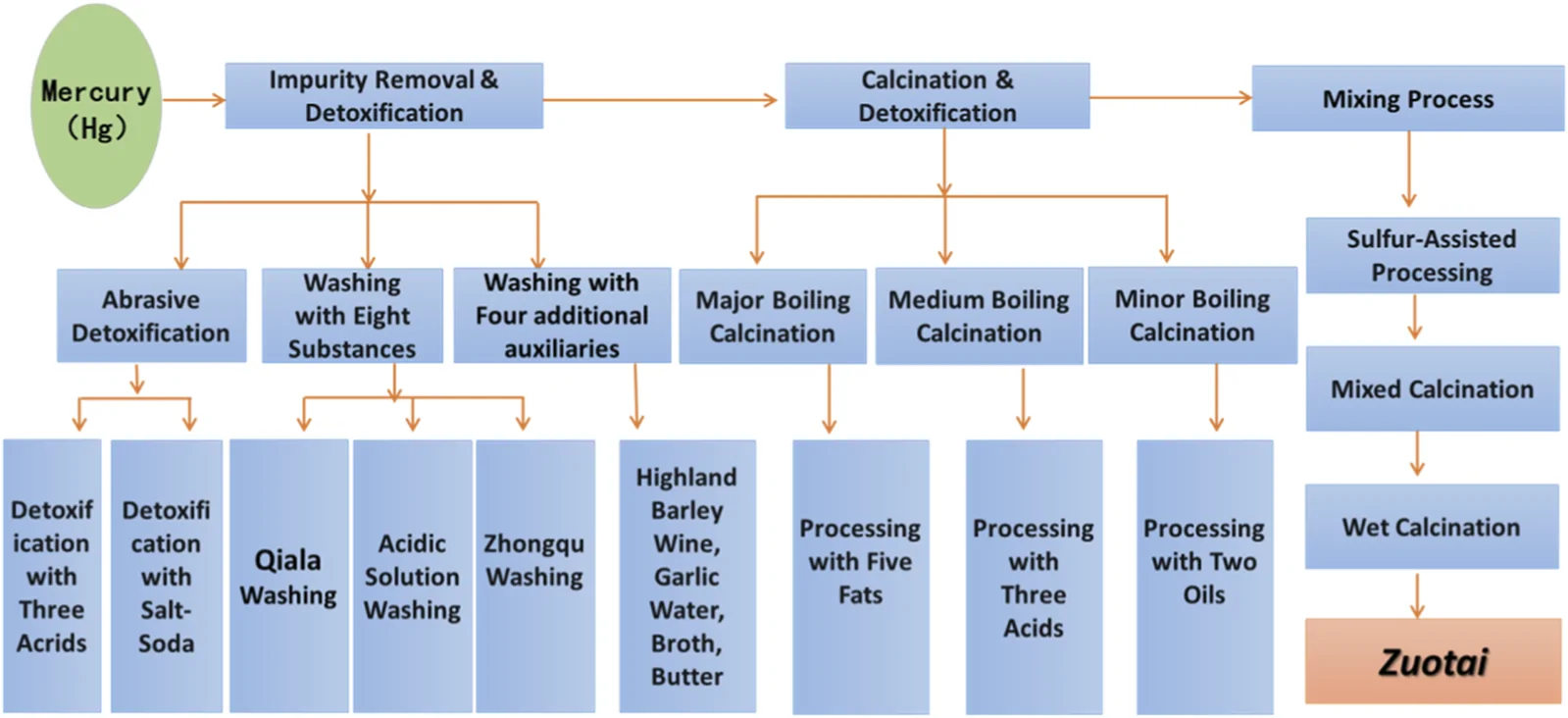
Technical flowchart for Zuotai processing.

The purified mercury then underwent a multi-stage calcination process. This intricate process consisted of three distinct boiling stages with various ingredients, followed by mixing process including sulfur-assisted processing, mixing and string, wet string, and finally, dry calcination. Each stage was meticulously controlled in terms of temperature, duration, and ingredient addition, following strict procedural protocols to transform the mercury into the final, therapeutically intended Zuotai preparation.

### 2.3 *In vitro* simulated gastrointestinal fluid dissolution test

#### 2.3.1 Chemicals and reagents

Hydrochloric acid (HCl, guaranteed grade, 36%–38%, Sinopharm), pepsin (from porcine gastric mucosa, 1:10,000 activity, Sigma-Aldrich), potassium dihydrogen phosphate (KH_2_PO_4_, analytical grade, Sinopharm), sodium hydroxide (NaOH, analytical grade, Sinopharm), pancreatin (from porcine pancreas, USP grade, Sigma-Aldrich), L-cysteine (analytical grade, ≥99%, Sinopharm), and nitric acid (HNO_3_, guaranteed grade, 65%, Sinopharm) were used. Gold standard solution (1000 μg/mL, Sigma-Aldrich) was added to all samples and standards at 50 μg/L to stabilize mercury and eliminate memory effects during ICP-MS analysis. Ultrapure water (18.2 MΩ•cm) was used throughout. All reagents were of analytical grade or higher and used without further purification.

#### 2.3.2 Dissolution procedure

The *in vitro* dissolution test was performed following the method of previous research (Ming et al., 2018). Simulated gastric fluid (SGF, pH 1.2) was prepared with pepsin (10 g/L) according to the Chinese Pharmacopoeia (2020 edition). Simulated intestinal fluid (SIF, pH 6.8) was prepared with pancreatin (10 g/L). L cysteine was added to selected groups at a final concentration of 10 mM to simulate physiological thiol conditions.

Zuotai samples were ground, sieved (200 mesh), and dried at 105 °C to constant weight. Each sample (0.25 g) was placed in a 50 mL conical flask with 25 mL of dissolution medium (solid-to-liquid ratio 1:100). The mixtures were incubated in a water bath shaker at 37 °C with 100 rpm agitation for 24 h. After incubation, the supernatants were collected by centrifugation (12,000 rpm, 10 min) and filtered through 0.22 μm membranes. The filtrates were acidified with 2% nitric acid and stabilized with gold solution (50 μg/L) to eliminate mercury memory effects.

Soluble mercury concentrations were determined by inductively coupled plasma mass spectrometry (ICP-MS, Agilent 7900) with bismuth (^209^Bi) as internal standard. Method parameters: RF power 1550 W, carrier gas flow 1.08 L/min, He collision mode. The limit of detection (LOD) for mercury was 0.01 μg/L. Methylmercury and ethylmercury were analyzed by HPLC-ICP-MS when applicable. All measurements were performed in triplicate.

## 3 Results and discussion

The present study characterized five batches of Zuotai samples prepared across different Tibetan medical institutions. The essential information of these samples, including their manufacturing sites and dates, is summarized in Table 3.

**TABLE 3:** Essential information of the five batches of Zuotai samples.

Manufacturing site	Sample ID	Production date
Affiliated Hospital, Tibet University of Tibetan Medicine	ZT001	May 2017
Diqing Tibetan Hospital, Yunnan	ZT002	June 2018
Gansu Kaja Tibetan Medicine Co., Ltd.	ZT003	June 2019
Yongzhong Tibetan Hospital Dingqing County, Tibet Autonomous Region	ZT004	June 2020
Hongyuan County Tibetan Hospital, Sichuan	ZT005	June 2023

### 3.1 Crystalline phase analysis of samples

The phase transitions and crystal structures of the samples were analyzed using an X-ray diffractometer (XRD, Bruker D8 Advance) with a scanning speed of 5°/min and a scanning range of 10°–90°. XRD analysis reveals the crystal structure of Zuotai’s components, which is crucial for understanding its therapeutic properties (Eskalen et al., 2021; Qin et al., 2023). The XRD patterns of the five samples are shown in Figure 2. All samples exhibit distinct characteristic peaks corresponding to HgS (PDF standard card #75–1538) and elemental sulfur S (PDF# 99-0066). As observed from the XRD patterns, characteristic peaks of HgS {Cubic, F-43m (216)} [PDF standard card #89-0432] were detected at 2θ = 26.3°, 30.5°, 43.7°, 51.8°, 54.2°, and 70.0°, etc. Meanwhile, characteristic peaks of elemental S {Orthorhombic, Fddd (70)} [PDF# 99-0100] were identified at 2θ = 15.3°, 23.0°, 25.8°, 31.4°, etc. No other crystalline phases were observed in any of the samples besides the aforementioned two phases. The XRD patterns are consistent with those reported in the literature (Xu et al., 2023). This indicates an excess of sulfur in the starting materials, and the heavy metal mercury (Hg) component fully reacted with sulfur to form well-crystallized HgS, which constitutes the primary phase in the samples.

**FIGURE 2:**
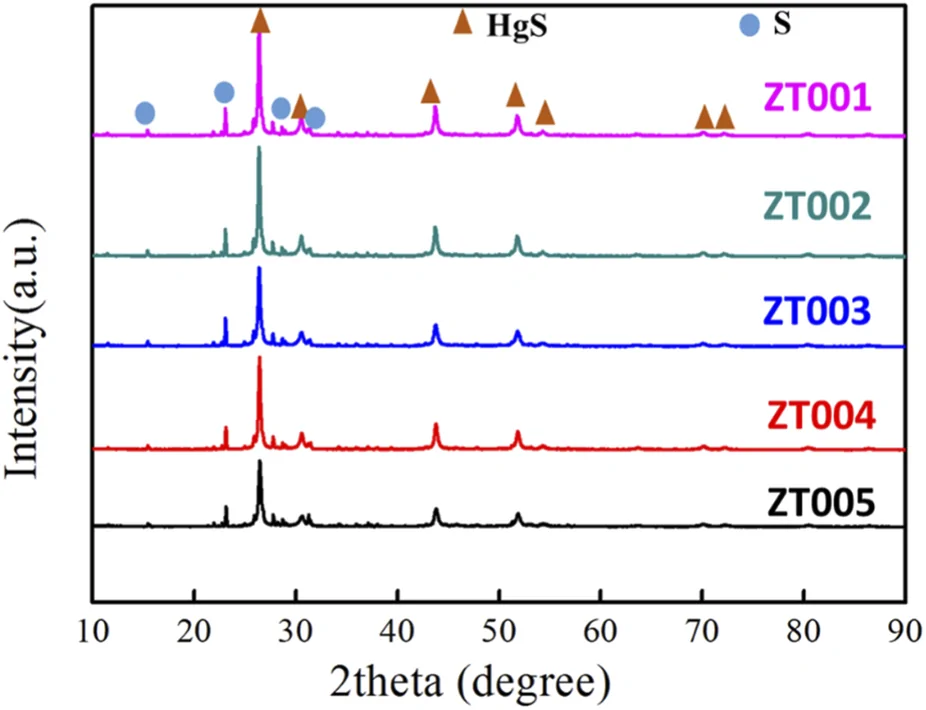
XRD patterns of the five batches of Zuotai samples.

To enable cross-comparison with traditionally processed Zuotai in the absence of physically archived samples, we compiled published characterization data from peer-reviewed studies that utilized traditional manual processing. Comparative analysis reveals that while both processing methods yield identical primary crystalline phases (HgS with excess S), the modern jacketed reactor method produces significantly improved batch-to-batch consistency, as evidenced by lower coefficients of variation across all major elemental components.

### 3.2 Microstructure analysis of the samples

SEM imaging was performed at an accelerating voltage of 5 kV with a working distance of 8.5 mm using the SE2 detector mode. The microstructure of all samples was examined using a cold-field emission scanning electron microscope (SEM, ZEISS Sigma 300), and the results are presented in Figure 3. All samples in this experiment exhibited similar structures. Figures 3a,b shows macroscopic images of the Zuotai sample, revealing that the obtained sample is a fine black powder. The powder particles are dispersed without agglomeration and possess a distinctive medicinal odor. When a small amount of the black powder was placed in a volume of water, it was observed to float on the water’s surface, indicating that the sample is insoluble in water. The true density, measured using a density analyzer (Micromeritics AccuPyc II 1340), was approximately 3.3855 g/cm^3^. Figure 3c1 display the microstructures of the five samples at different magnification levels. It can be observed that the Zuotai samples exhibit a microstructure composed of clustered spherical nanoparticles. The particle size is approximately 100–200 nm, and these small nanoparticles assemble through stacking to form larger particles. The overall structure exists in an aggregated state, which ensures a relatively stable internal framework within the Zuotai material.

**FIGURE 3:**
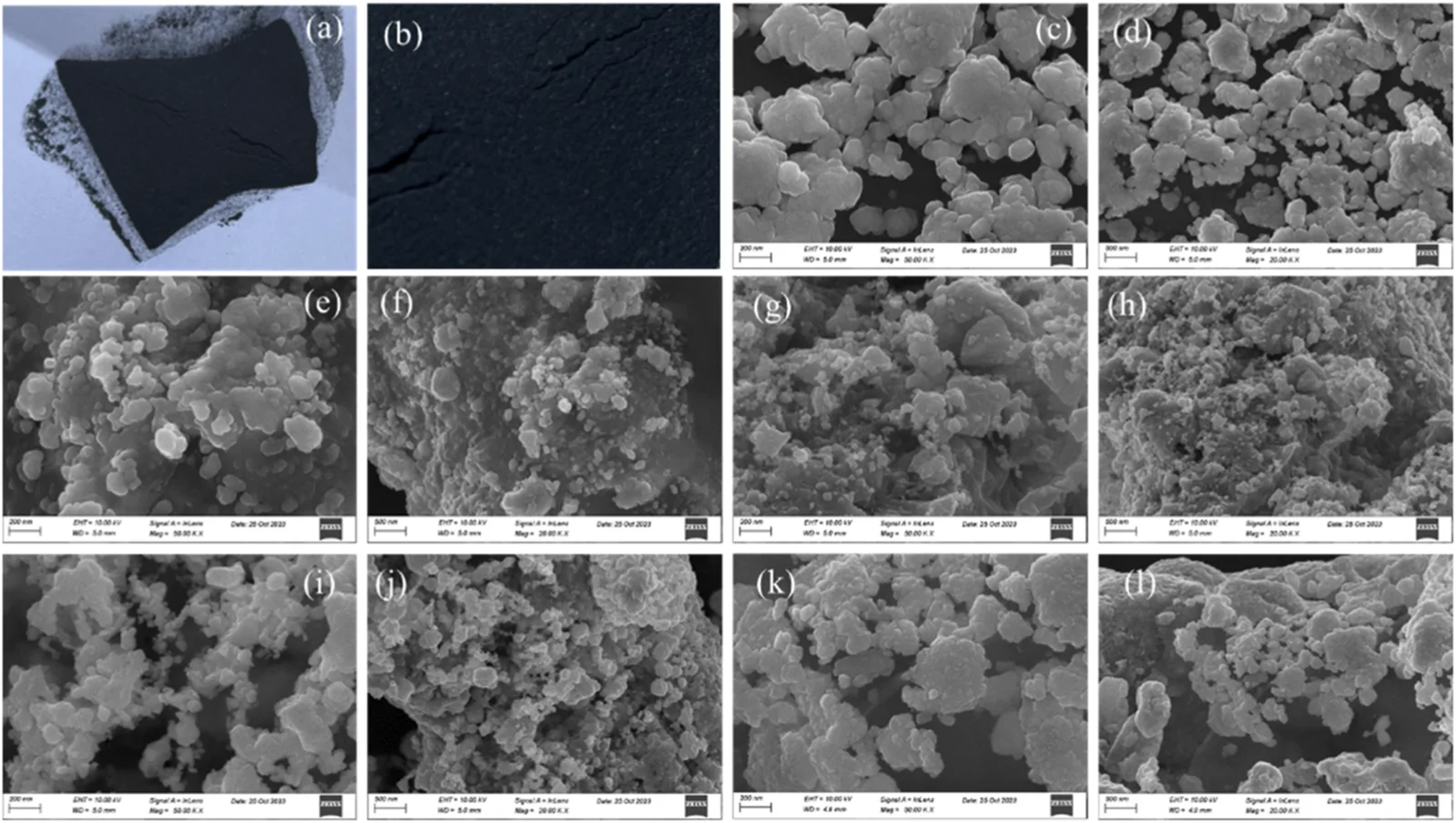
Macrographs and SEM images of the Zuotai sample: **(a,b)** Macrographs of the Zuotai sample; **(c-l)** SEM images of the five sample batches at different magnification levels.

Taking sample ZT003 as an example, we analyzed the overall particle size distribution of the nanomaterials using Dynamic Light Scattering (DLS). DLS measurements were performed at 25 °C with a 173° scattering angle. Data are reported as Z-average ±standard deviation from three replicate measurements. The sample was dissolved in ethanol, oscillated for 3 min, sonicated for 10 min, and then oscillated again for 1 min. Immediately after, Dynamic Light Scattering (DLS) was used to analyze the particle size distribution of the small particles dispersed in ethanol solvent, and the results are shown in Figure 4. The size of the small particles of this sample dispersed in ethanol solvent is 164 ± 49 nm.

**FIGURE 4:**
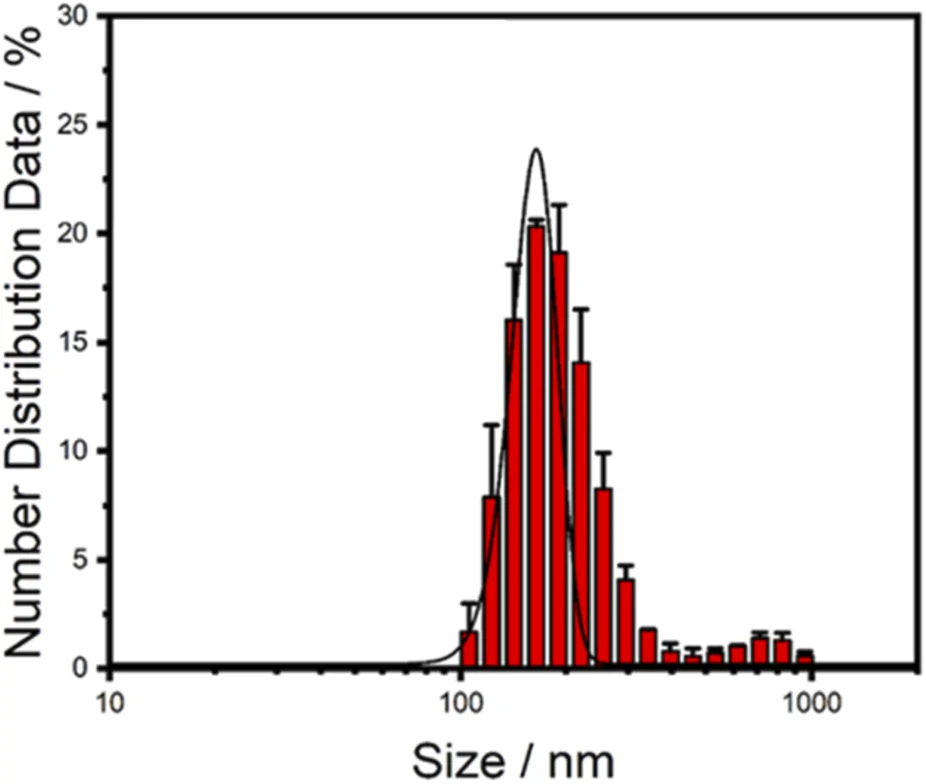
Overall particle size distribution of the ZT003 sample.

Meanwhile, we analyzed the morphology and main elemental composition of the samples via SEM-EDS point scanning. As shown in Figure 5, the nanoparticles in the sample form nanoclusters through mutual stacking, and Hg and S are uniformly dispersed throughout the nanocluster structure. From the EDS point scanning data, the atomic ratio of Hg to S is less than 1, indicating an overall excess of S, which is consistent with the initial raw material addition. Similar results were observed for other samples (see Table 4).

**FIGURE 5:**
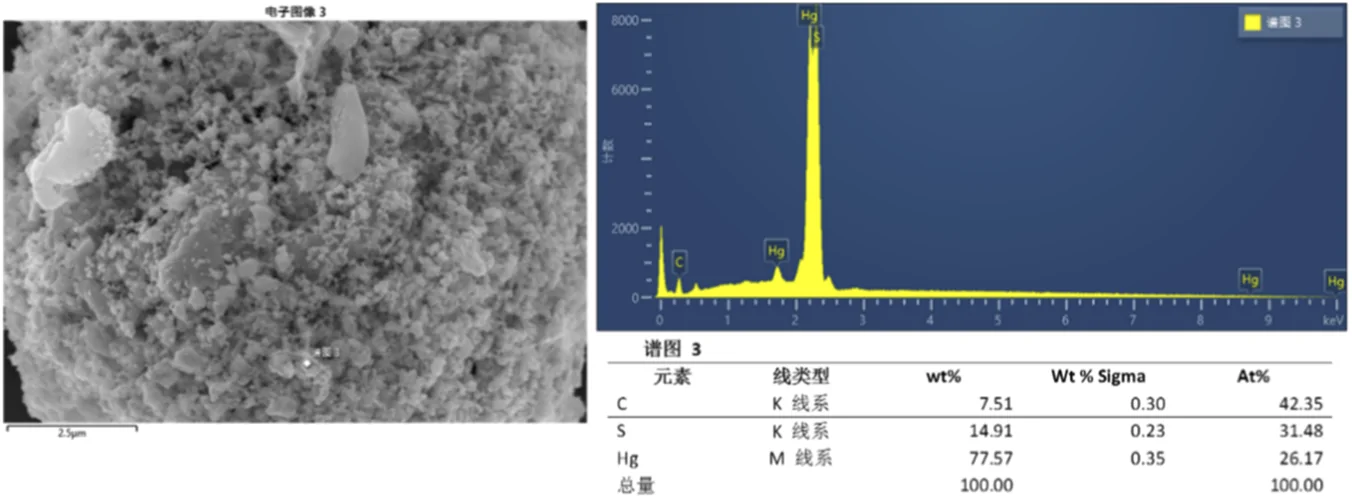
EDS point analysis of the ZT003 sample.

**TABLE 4:** EDS elemental analysis (atomic %) of the five batches.

Atomic %	ZT001	ZT002	ZT003	ZT004	ZT005
C K	28.22	33.67	42.35	33.67	46.38
S K	37.21	36.19	31.48	36.69	27.40
Hg K	34.57	29.64	26.17	29.64	26.22
Totals	100.00	100.00	100.00	100.00	100.00

Statistical consistency: The coefficient of variation (CV) for Hg content across five batches was 11.3%, and for S content 11.8%, indicating acceptable batch-to-batch reproducibility for a complex Tibetan medicine.

### 3.3 Elemental content analysis

Qualitative and quantitative elemental analysis of the Zuotai powder sample was performed using X-ray fluorescence spectroscopy (XRF, ZSX Primus III+), with the composition expressed as mass percentages of the elements. As summarized in Table 5, sulfur (S) and mercury (Hg) were identified as the predominant components, accounting for approximately 80 mass% and 15 mass%, respectively. The sample also contained other elements, including Mg, Al, Si, K, Ca, Fe, Cu, Zn, Sn, V, Mn, Ag, and Pb, which represent the principal elemental constituents of the “Eight Metals and Eight Mineral Ashes.” According to the analytical results, the combined content of S and Hg reached about 95 mass%. The “Eight Metals” fraction constituted approximately 0.5 mass%, while other rare earth elements accounted for about 0.6 mass%, and the remaining components totaled roughly 4.0 mass%. The overall composition sums to nearly 100 mass%, as illustrated in Figure 6. One-way ANOVA revealed no statistically significant differences (p > 0.05) for any of the detected elements (including Hg, S, Pb, Cu, Ag, Fe, Mn, Zn, Ca, and Mg) across the five production batches. These results confirm that the modern jacketed reactor process yields consistent product quality, which is essential for clinical application and regulatory acceptance.

**TABLE 5:** Elemental distribution in the Zuotai samples.

Element	ZT001 (wt%)	ZT002 (wt%)	ZT003 (wt%)	ZT004 (wt%)	ZT005 (wt%)
MgO Al_2_O_3_ SiO_2_ P_2_O_5_ SO_3_ K_2_O CaO V_2_O_5_ MnO Fe_2_O_3_ CuO Ag_2_O SnO_2_ ZnO HgO PbO ……	0.1161 0.1088 0.2211 0.0085 83.5791 0.1031 0.1220 0.0517 0.0433 0.2470 0.1094 0.0315 0.0216 0.0251 15.1063 0.0759	0.2431 0.1215 0.2241 0.1627 81.5438 0.1180 0.1015 0.0550 0.0453 0.2625 0.1253 0.0349 0.0403 0.0648 16.7662 0.0908	0.3239 0.1780 0.3583 0.0305 82.4966 0.1729 0.1123 0.0435 0.0452 0.3621 0.1503 0.0617 - 0.0748 15.5702 0.0195	0.3303 0.1963 0.3797 0.0227 81.8628 0.1816 0.1226 0.0511 0.0515 0.3721 0.1505 0.0618 - 0.0706 15.1076 0.0181	0.0788 0.0776 0.1637 0.0202 82.3470 0.0614 0.0461 0.0478 0.0484 0.1943 0.0744 - 0.0270 - 16.7324 0.0810

**FIGURE 6:**
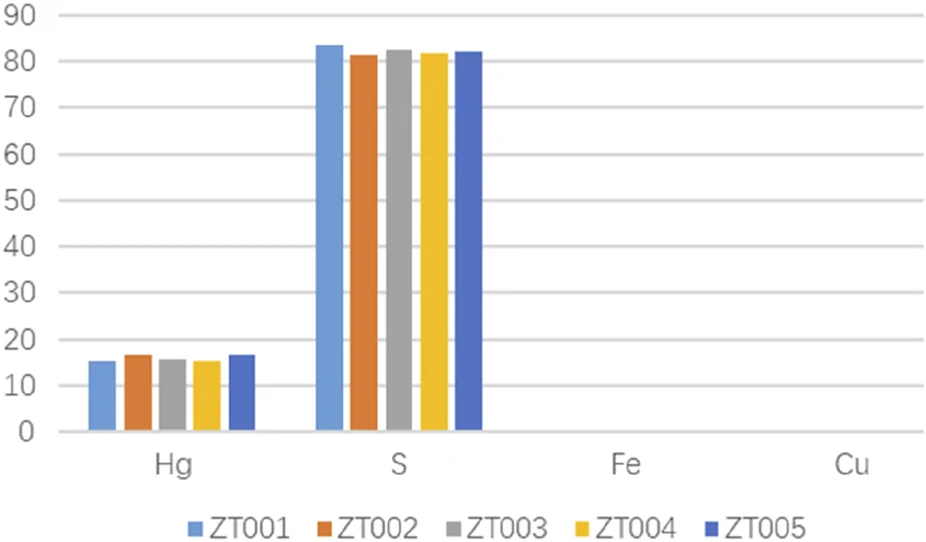
Major elemental distribution in the five batches of Zuotai samples.

### 3.4 Functional group analysis by FT-IR

Infrared spectroscopy can provide information about chemical bonds and functional groups in samples. FT-IR spectra were recorded at 4 cm^-1^ resolution with 64 scans accumulation; background spectra were obtained with KBr pellets over the range 400–4000 cm^-1^. The main component of Zuotai samples is inorganic substances such as HgS, but many plant and animal medicinal materials are added during the processing of Zuotai, so the samples may contain small amounts of organic substances (Xiaoyan et al., 2013). However, the organic components added during processing are too complex to be analyzed in detail. Some literatures have reported the observation of certain organic compounds by high-performance liquid chromatography. In this paper, we used infrared spectroscopy to preliminarily explore the possible organic components in the samples, and the results are shown in Figure 7. A broad band at 3363 cm^-1^ indicates the presence of–OH or–COOH; the peak around 1620 cm^-1^ is assigned to the C=O bond; and the peak at approximately 717 cm^-1^ may correspond to the C–H bond on aromatic rings.

**FIGURE 7:**
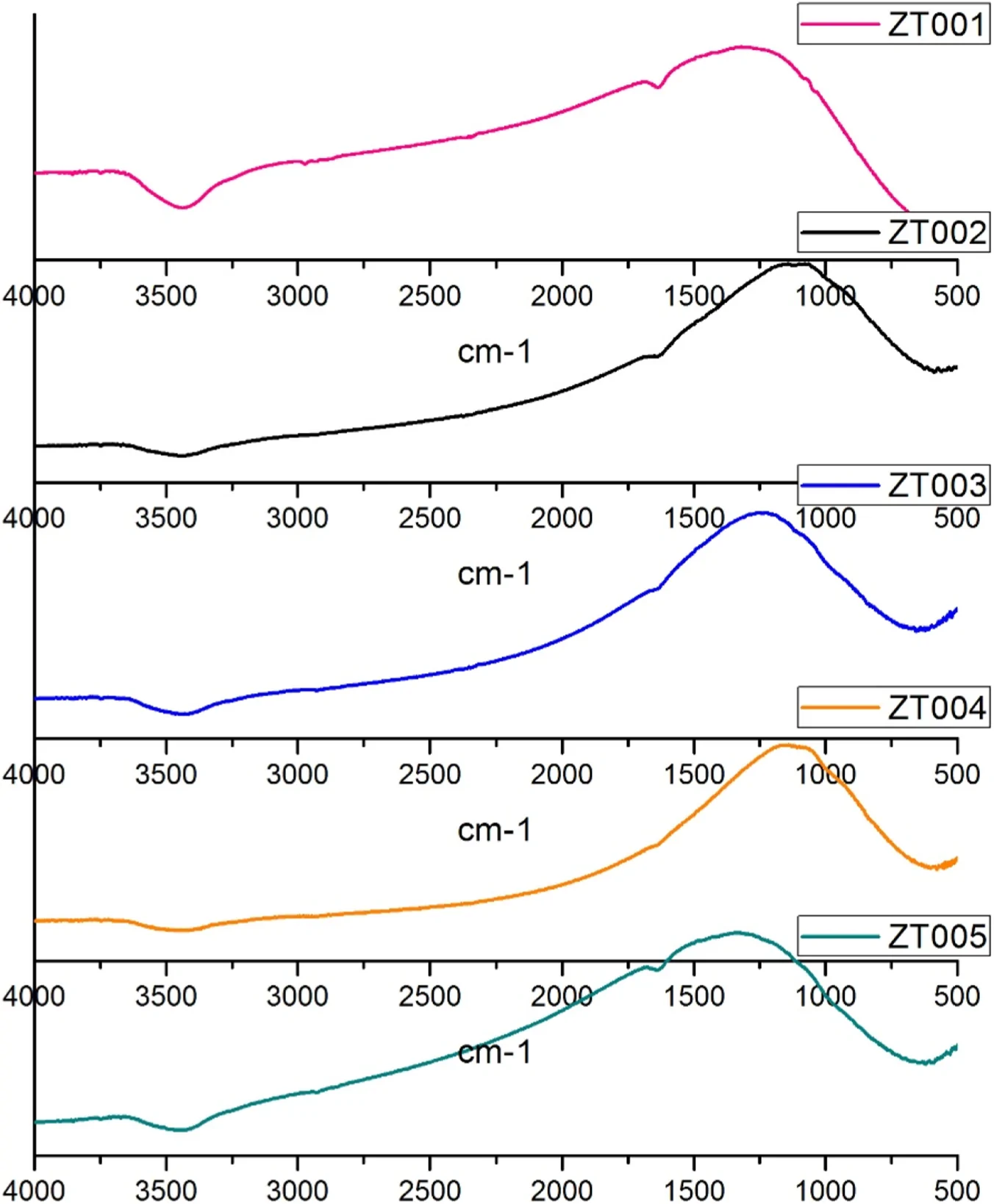
FT-IR spectra of the five batches of Zuotai samples.

The weak FT IR bands at 3363, 1620, and 717 cm^-1^ suggest the presence of trace organic residues derived from plant and animal auxiliaries. However, at high calcination temperatures, most organic matter undergoes carbonization, leaving only minor oxygen- and nitrogen-containing functional groups in the final product. Previous studies using HPLC, UPLC-MS, and NMR have also been unable to definitively identify these trace constituents (Gaowu et al., 2010). Comprehensive identification by GC-MS or HPLC is beyond the current scope but remains a direction for future investigation.

### 3.5 Mercury speciation and dissolution safety assessment

The mercury speciation analysis by sequential extraction (modified Tessier method) revealed that >99.6% of total mercury in modern-processed Zuotai existed in the residual HgS fraction, with exchangeable Hg^2+^ below the ICP-MS detection limit (0.01 μg/L), confirming that the vast majority of mercury is present as the insoluble, biologically inert HgS phase.

The *in vitro* dissolution results are summarized in Table 6. Under simulated gastric fluid (SGF, pH 1.2) without L-cysteine, the soluble mercury release from modern-processed Zuotai was 19.71 μg/g (0.0131% of total Hg). Addition of 10 mM L-cysteine-a physiologically relevant thiol compound that enhances mercury complexation-increased the release to 38.64 μg/g (0.0258% of total Hg). In simulated intestinal fluid (SIF, pH 6.8), the corresponding values were 2.54 μg/g (0.00169%) without L-cysteine and 34.58 μg/g (0.0231%) with L-cysteine. The negative control (ultrapure water) showed only 1.90 μg/g (0.00127%), confirming that dissolution is driven by the digestive environment rather than inherent solubility of the material.

**TABLE 6:** *In vitro* soluble mercury release from Zuotai in simulated gastrointestinal fluids (ICP-MS, 24 h, 37 °C).

Sample	Dissolution medium	Net soluble Hg (mg/L)	Net soluble Hg (μg/g Zuotai)	Dissolution rate (% of total Hg)
Modern Zuotai	SGF (pH 1.2)	0.1971	19.71	0.0131%
Modern Zuotai	SGF + L-Cys (10 mM)	**0.3864**	**38.64**	**0.0258%**
Modern Zuotai	SIF (pH 6.8)	0.0254	2.54	0.00169%
Modern Zuotai	SIF + L-Cys (10 mM)	0.3458	34.58	0.0231%
Modern Zuotai	Ultrapure water (pH 7.0)	0.0190	1.90	0.00127%
Traditional Zuotai	SGF + L-Cys (10 mM)	0.1060	10.60	0.00707%

Total Hg in Zuotai samples = approximately 15 wt% (150,000 μg/g) by XRF. Values are net of reagent blank (0.0348 mg/L). LOD, 0.01 mg/L.

A traditional-process Zuotai sample, analyzed under the same conditions (SGF + L-cysteine), showed a soluble mercury release of 10.60 μg/g (0.00707% of total Hg), confirming that the modern process maintains the intrinsic low-toxicity profile of traditional Zuotai.

Importantly, HPLC-ICP-MS analysis confirmed that no methylmercury or ethylmercury species were detected in any of the dissolution supernatants, consistent with previous findings (Bu et al., 2022). Based on the maximum soluble mercury release (38.64 μg/g) and the typical clinical dose of Zuotai-containing formulations (approximately 0.2 g per day), the estimated daily intake of soluble mercury is approximately 7.73 μg—only 18% of the FAO/WHO acceptable daily intake (ADI) for mercury (43 μg/day for a 60 kg adult).

Comparison with literature data (Ming et al., 2018) demonstrates that while traditional Zuotai samples show up to 120-fold variation in mercury dissolution across 16 batches, our five modern-process batches exhibit no statistically significant batch-to-batch variation (CV < 12%, p > 0.05 by one-way ANOVA), confirming the superior consistency of the jacketed reactor method.

## 4 Conclusion

In the traditional production of Tibetan medicine Zuotai, the contradiction between primitive equipment, small production scale, and the growing market demand for high-quality products has become increasingly prominent. Therefore, the traditional manual processing technology urgently requires innovation. The Gawe’ research team adopted a novel reaction method using an electric-heated stainless steel jacketed pot, improving the traditional manual techniques such as leather bag kneading and stone mortar grinding. By analyzing and controlling chemical and physical parameters including raw material ratios, temperature, and humidity, the previously intractable processing technology has been transformed into a controllable, replicable, promotable, and highly operable routine technique.

The 100–200 nm HgS nanoclusters observed in modern-processed Zuotai samples (164 ± 49 nm by DLS) are consistent with the size range of biosynthesized HgS nanoparticles reported in the literature. However, the direct correlation between this nanostructure and *in vivo* pharmacological activity—including absorption, biodistribution, and therapeutic efficacy—requires dedicated investigation and constitutes a priority direction for our future research.

Compared to the traditional processing method, this approach significantly reduces the processing time, lowers costs in material sourcing, workflow, and on-site experimentation, effectively mitigates health risks to operators during the process, and saves human, material, and financial resources. It is characterized by high efficiency, safety, and quality assurance. The safety of modern-processed Zuotai was further validated by *in vitro* dissolution testing. Under the most stringent simulated digestive conditions, the soluble mercury release was only 38.64 μg/g (0.0258% of total Hg), corresponding to an estimated daily intake of 7.73 μg—only 18% of the FAO/WHO acceptable daily intake (43 μg/day). No organic mercury species (methylmercury or ethylmercury) were detected in any dissolution supernatants. These results confirm that the modern jacketed reactor process effectively stabilizes mercury in its biologically inert HgS form, ensuring the safety of the final product.

The five batches of samples involved in this study exhibited consistent physicochemical properties and have been widely used in the clinical preparation of Tibetan medicines, demonstrating the innovativeness and scalability of this technology. Guided by traditional theory and processing sequences, our team’s preparation method prioritizes convenient, efficient, energy-saving, and safe techniques. This achievement meets the goals of shorter time, lower cost, stable parameters, controllable quality, and enhanced safety, providing significant technical guidance for the standardization and normalization of Zuotai processing in Tibetan medicine.

## Data Availability

The original contributions presented in the study are included in the article/supplementary material, further inquiries can be directed to the corresponding authors.

## References

[B1] Bowman B. A. Russell R. M. (2006). Present Knowledge in Nutrition[M]. Washington, DC: International Life Sciences Institute.

[B10] Bu H. L. Y. Qunpei Junrong, L. Mintao J. Yang W. Quan M. (2022). Speciation and valence analysis of mercury and arsenic in Tibetan medicine Zuotai by HPLC-ICP-MS and safety evaluation. Chin. Traditional Herb. Drugs, 53 (10), 2985–2990.

[B17] Cairen B. Maoji Z. Lianping D. Cairang N. Duojie A. (2022). Investigation on the current state of inheritance of the processing techniques of Tibetan medicine “zuotai”. Qinghai Sci. Technol. 29 (1), 66–69.

[B2] Cen L Ji D. Dui Z. Zhenjiang X. Yuzhi D. Lixin W. (2012). Chemical composition and structural analysis of the ash of “neng chi Ba jin”, the raw material for the refining formula of Tibetan medicine zuotai. China J. Chin. Materia Medica 37 (13), 1952–1957. 10.4268/cjcmm20121319 23019877

[B3] Chinese Intangible Cultural Heritage: Tibetan Medicine[N] (2008). Beijing, China: World News World Daily.

[B4] Cuo R. C. L. (2001). Detailed Explanation of the Four Medical Tantras (Volume 6)[M]. Beijing: Minzu Publishing House.

[B5] Dun Man X. N. J. C. (2011). “Mercury processing classics[M],” in Qinghai Institute of Tibetan Medicine Compilation. Tibetan Medicine Canon (Beijing: Minzu Publishing House), 58, 419.

[B6] Eskalen H. Çeşme M. Kerli S. Özğan Ş. (2021). Green synthesis of water-soluble fluorescent carbon dots from rosemary leaves: applications in food storage capacity, fingerprint detection, and antibacterial activity. Chem. Res. 45, 428–435. 10.1177/1747519820953823

[B7] European Medicines Agency (2022). “Guideline on quality of herbal medicinal products/traditional herbal medicinal products (revision 3)[M],” 3. London: EMA.

[B8] Ga D. (2005). Analyzing the toxicity of Tibetan Pharmacist-prepared medicine in “Zuoai” as Wess as its compatibility. Coll. Educ. Sci. Ed. 4, 72.

[B13] Gaowu L. Zhongjie C. Wenhu L. Kezhong W. Jian C. Xianglan P. (2010). Component analysis of Tibetan medicine “Zuotai”. Lishizhen Med. Materia Medica Res. 21 (12), 3209–3211.

[B9] Gyetsen T. Namdro T. Mikmar A. (2024). Inheritance and development of Tibetan medicine in the new era—an interview with prof Gawai Doji, a famous expert in Tibetan medicine and national intangible cultural Heritage inheritor. J. Xizang Univ. 39(3): 1–7. 10.16249/j.cnki.1005-5738.2024.03.001

[B11] He Z. Song G. Wang F. (2015). Textual research on the origin of the production techniques of Tibetan medicine “Zuotai”. Chin. Med. Cult. 10 (1), 47–50. 10.16307/j.1673-6281.2015.01.019

[B12] JianCuo S. RuoYu G. ChengJin L. Bing Z. Zhijian L. (2025). Bibliometric Study and pharmacovigilance considerations of Tibetan medicine zuotai. Chin. J. Ration. Drug Use 18 (15), 34–39. 10.15887/j.cnki.13-1389/r.2025.15.009

[B14] Lang S. (2007). A brief introduction to chronomedicine in Tibetan medicine. J. Med. and Pharm. Chin. Minorities 13 (5), 40. 10.16041/j.cnki.cn15-1175.2007.05.039

[B15] Luo D. (1986). New Repair Jingzhu Materia Medica (Jingzhu Bencao, Chinese Version)[M]. Shanghai: Shanghai Science and Technology Press.

[B16] Luo S. D. J. (2000). Zuotai Refining Secret[M]. Xianyang: Xianyang Wendu Printing Co., Ltd.

[B33] Ming Z. Hongtao B. Cen L. Yuzhi D. Lixin W. (2018). Differences in mercury dissolution of Tibetan medicine Zuotai in simulated gastric and intestinal fluids. Chin. Tradit. Pat. Med. 40 (10), 2302–2304.

[B24] Mingcheng W. Jingshu L. Yuwen Q. (2009). Application of rannasangpei in the treatment of cardiovascular and cerebrovascular diseases. Pharm. Care Res. 9 (4), 283–285.

[B18] National Pharmacopoeia Commission (2020). Pharmacopoeia of the People's Republic of China. Beijing: China Medical Science and Technology Press.

[B19] Ni Ma C. R. (2017). Tibetan Medicine Mercury Refining Method[M]. Lhasa: Tibet Tibetan Ancient Books. Lhasa, China: Tibet Tibetan Ancient Books Publishing House.

[B20] Nima G. (2024). The Essential Writings of Master Gawu, A Renowned Tibetan Physician[M]. Chengdu: Sichuan Nationalities Publishing House.

[B25] Qin W. Nina W. Luwei X. Huilian H. Jindong L. Yong J. (2023). TCM fingerprint database: a digital approach to scientifically reflect the internal quality of traditional Chinese medicine. Pharmacol. Res. - Mod. Chin. Med. 7 (2023), 100261. 10.1016/j.prmcm.2023.100261

[B21] Qunpei Wenbing, L. Dan Duoji C. D. Yuan L. Zhuoma D. (2025). Historical evolution and modern research progress of zuotai processing. Chin. Tradit. Pat. Med. 47 (4), 1252–1258.

[B22] Rang D. J. C. Yingfeng M. Lijuan W. Cairen B. (2025). Textual research on the similarities and differences and origins of the detoxification processing methods between Tibetan medicine “Zuotai” and Mongolian Medicine “Menggen Wusu”. Guid. J. Traditional Chin. Med. Pharm. 25 (13), 34–35. 10.13862/j.cnki.cn43-1446/r.2019.13.010

[B23] U.S. Food and Drug Administration (2016). Botanical Drug Development: Guidance for Industry [M]. Silver Spring, MD: FDA.

[B26] Wu Q. Wen-Kui Li Zhou Z. P. Li Y. Y. Xiong T. W. Du Y. Z. (2016). The Tibetan medicine zuotai differs from HgCl2 and MeHg in producing liver injury in mice. Regul. Toxicol. Pharmacol. 78, 1–7. 10.1016/j.yrtph.2016.03.017 27032305

[B27] Xiangyang L. WenLi F. Qisheng M. Junbo Z. Cairang N. Li G. (2010). Preliminary study on the absorption and excretion of Mercury in Tibetan medicine Zuotai in rats. Lishizhen Med. Materia Medica Res. 21 (2), 290–292.

[B28] Xiaoyan Z. Mei S. Jingxia W. Yunzhang X. Yuan L. Zhifeng Z. (2013). Characterization of Tibetan medicine Zuota by multiple techniques. Bioinorganic Chem. Appl. 198545, 1–11. 10.1155/2013/198545 PMC3777212 24093005

[B29] Xu D. Yuan C. Tang J. (2023). Study on electrochemical and X-ray diffraction fingerprint of Tibetan medicine “zuotai”. Int. J. Electrochem. Sci. 18, 100259. 10.1016/j.ijoes.2023.100259

[B30] Yan L. Ma X. Zhu Q. (2007). The analysis of inorganic components in Tibetan medicine ZuoTai. China J. Chin. Materia Medica 32 (2), 159.

[B31] Yutuo Yuandan G. (1987). The Four Tantras (Of Medicine)[M]. Shanghai, China: Shanghai Science and Technology Publishers, 280.

[B32] Zhang Y. Xiang Y. (2005). To analyze the usage of toxicant in traditional Tibetan medicine based on 64 dermatological formulas by oral administration. World Sci. Technology/Modernization Traditional Chin. Med. Materia Medica 7, 64–66.

